# Studying the local character of Raman features of single-walled carbon nanotubes along a bundle using TERS

**DOI:** 10.1186/1556-276X-6-174

**Published:** 2011-02-25

**Authors:** Niculina Peica, Christian Thomsen, Janina Maultzsch

**Affiliations:** 1 Institut für Festkörperphysik, Technische Universität Berlin, Hardenbergstr. 36, 10623 Berlin, Germany

## Abstract

Here, we show that the Raman intensity of the G-mode in tip-enhanced Raman spectroscopy (TERS) is strongly dependent on the height of the bundle. Moreover, using TERS we are able to position different single-walled carbon nanotubes along a bundle, by correlating the observed radial breathing mode (RBM) with the AFM topography at the measuring point. The frequency of the G^- ^mode behaves differently in TERS as compared to far-field Raman. Using the RBM frequency, the diameters of the tubes were calculated and a very good agreement with the G^-^-mode frequency was observed.

## Introduction

Tip-enhanced Raman spectroscopy (TERS) became a very useful technique in studying the optical properties of carbon nanotubes [1-6]. A previous study [7] on the detection of single-walled carbon nanotubes (SWCNT) by using TERS concentrated on showing the G-mode and the radial breathing mode (RBM) from a nanometer-sized region that could not be visible in the micro-Raman measurements. Another report [8] has focused on studying the variations in the Raman spectra of the G-mode and RBM by changing the polarization conditions and has shown different behaviors for the two distinct modes. Identification of the chiral indices of SWCNT through the observed radial breathing mode (RBM) in near-field Raman and photoluminescence (PL) of the nanotubes was reported as well [9]. Combining near-field PL and near-field Raman imaging, Hartschuh et al. [10] observed higher near-field enhancements using PL and suggested that using these two techniques for the study of individual SWCNTs it should be possible to correlate the structural defects with the emission properties of the nanotubes. Recently, Roy and Williams [11] developed a new spectrometer for high resolution Raman imaging of SWCNTs and showed TERS images of SWCNTs using radially polarized circular and annular beams, respectively. In order to extract structural information from SWCNTs in bundles we combine the AFM topography with the TERS and confocal Raman measurements along an SWCNTs bundle. The purpose of our study is to analyze the vibrational properties of SWCNTs along a bundle using TERS. From the observed RBMs in the TERS spectra and the extracted information from the AFM topography we attribute each RBM to a nanostructure from the measured bundle. Moreover a correlation of the diameter-dependent G^- ^peaks to the assigned RBMs is discussed.

### Experimental

The TERS measurements were performed using a commercially available combination of an AFM/STM XE-100 from Park Systems and a LabRam HR-800 spectrometer from Horiba Jobin Yvon. For excitation, the 532.2 nm line from a doubled-frequency Nd:YAG laser was used. The spectra were collected in backscattering geometry with a resolution of 2 cm^-1 ^and recorded with a Peltier-cooled CCD camera. The laser power on the sample used in our measurements was 0.1 mW. The TERS experiments were done in contact-mode AFM with an Au-coated tip. The silicon nitride AFM tips with a reflective Au-coating of 60 nm were purchased from Veeco and were coated with an extra 20 nm Au by thermal evaporation in a vacuum chamber kept at a pressure of 10^-5 ^mbar. SWCNTs were produced by high-pressure gas-phase decomposition of CO (HipCO), deposited on a Si/SiO_2 _substrate.

## Results and discussion

**Figure 1:**
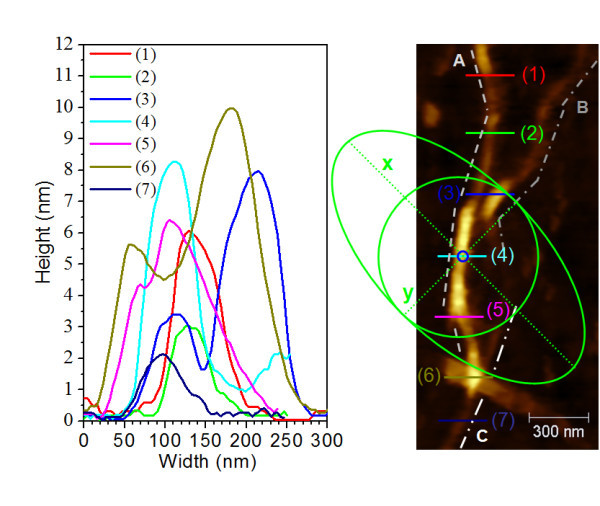
**Left-hand side: Height profiles of the seven measured positions along an SWCNTs bundle**. Right-hand side: AFM topography together with an approximation of the far-field spot area (green ellipse), diffraction-limited area (green circle), the near-field area (small blue circle), and the *y*-polarization direction of the incident laser. **(*A*)**, **(*B*)**, and **(*C*) **denote the three different bundles observed in the AFM topography. This notation is used in the RBM discussion part.

We performed TERS and confocal Raman measurements at seven different positions along a small SWCNTs bundle in order to study the local character of different Raman features of SWCNTs. The seven positions along the SWCNTs bundle are depicted in Figure 1 together with their corresponding AFM height profiles. Figure 1 shows the optically excited areas in the far-field (green ellipse) as well as in the near-field (blue circle). The tip-induced enhancement is coming from the small excited area in the near-field (Figure 1, blue circle) whereas the total signal in TERS always includes the confocal Raman signal, coming from the same area as in the far-field (Figure 1, green ellipse) and thus including more carbon nanostructures than the near-field area. The incident laser is coming under an angle of 60° with respect to the surface normal and its polarization direction is depicted in Figure 1 by the *y*-axis of the green ellipse.

The heights and the full widths at half maxima (FWHM) of the peaks after a Lorentzian fit of the height profiles are summarized in Table 1. The determined FWHMs are between 21 and 94 nm and the heights range from 2 to 9 nm. The height profiles indicate the presence of an SWCNT bundle.

**Table 1:** Heights and FWHM after a Lorentzian fit of the profiles at each measured position

Position	Height 1 (nm)	Height 2 (nm)	FWHM 1 (nm)	FWHM 2 (nm)	Cumulated FWHM (nm)
**1**.	6.80*		61.37		61.37
**2**.	3.40*		49.28		49.28
**3**.	2.70	8.30*	34.58	76.56	111.14
**4**.	9.00*		55.61		55.61
**5**.	1.80	6.40*	21.71	84.82	106.53
**6**.	4.40*	10.00	47.43	93.98	141.41
**7**.	2.22*		53.69		53.69

The star-marked height profiles correspond to the tip position, and were used for plotting the Raman signal dependence as a function of the height.

### Radial breathing modes

In the RBM region of the TERS spectra three or four different RBMs are observed at each of the marked positions (Figure 1). In total seven different RBMs are observed confirming the presence of an SWCNT bundle. In Table 2 we summarize all observed RBMs together with a tentative chiral-index assignment. Taking into account the observed RBM frequencies and the presence of bundled SWCNTs, the tubes' diameters were calculated [12] and based on previous theoretical studies [13] a tentative assignment of the chiral indices is also given (Table 2).

**Table 2:** Summary of the RBM frequencies in the TERS spectra along the measured bundle together with their calculated diameter and tentative chiral indices assignment

Position	**ω**_**RBM_1 **_ **(cm**^**-1**^**)** **d**_**t**_**_**_**1**_**(nm)**	**ω**_**RBM_2 **_ **(cm**^**-1**^**)** **d**_**t**_**_**_**2**_**(nm)**	ωRBM_3 **(cm)**^**-1**^ **d**_**t**_**_**_**3**_**(nm)**	**ω**_**RBM_4**_ **(cm)**^**-1**^ **d**_**t**_**_**_**4**_**(nm)**	**ω**_**RBM_5**_ **(cm)**^**-1**^ **d**_**t**_**_**_**5**_**(nm)**	**ω**_**RBM_6 **_ **(cm)**^**-1**^ **d**_**t**_**_**_**6**_**(nm)**	**ω**_**RBM_7 **_ **(cm)**^**-1**^ **d**_**t**___**7**_**(nm)**
**1**.	150.1 wm (1.63)	-	177.4 m (1.35)	-	-	-	277.9 m (0.83)
**2**.	150.1 wm (1.63)	-	178.1 wm (1.35)	-	-	-	277.1 ms (0.83)
**3**.	-	155.7 wm (1.56)	-	181.5 wm (1.32)	204.9 vw (1.15)	-	277.5 vs (0.83)
**4**.	-	-	-	182.7 m (1.31)	205.3 w (1.15)	-	276.9 s (0.83)
**5**.	-	-	-	183.3 ms (1.30)	205.7 w (1.15)	-	277.2 m (0.83)
**6**.	-	-	-	182.8 m (1.31)	-	244.2 wm (0.95)	276.9 wm (0.83)
**7**.	-	-	-	181.8 w (1.32)	-	244.1 w (0.95)	279.7 w (0.82)

**(n,m)** **E**_**ii**_**(eV)**	(17,6)^S^ 2.030	(20,0)^S^ 2.035	(13,6)^S^ 2.014	(15,3)^M^ 2.764	(11,5)^M^ 2.003	(10,3)^S^ 2.581	(8,4)^S^ 2.880
	*(12,12)*^*M*^ *2.539*	*(17,5)*^*M*^ *2.436*	*(14,5)*^*M*^ *2.763*	(12,7)^S^ 2.251	*(14,1)*^*S *^ *2.178*	*(12,0)*^*M*^ *1.934*	*(10,1)*^*M*^ *2.100*

w, weak; m, medium; s, strong; S, semiconducting; M, metallic.

**Figure 2:**
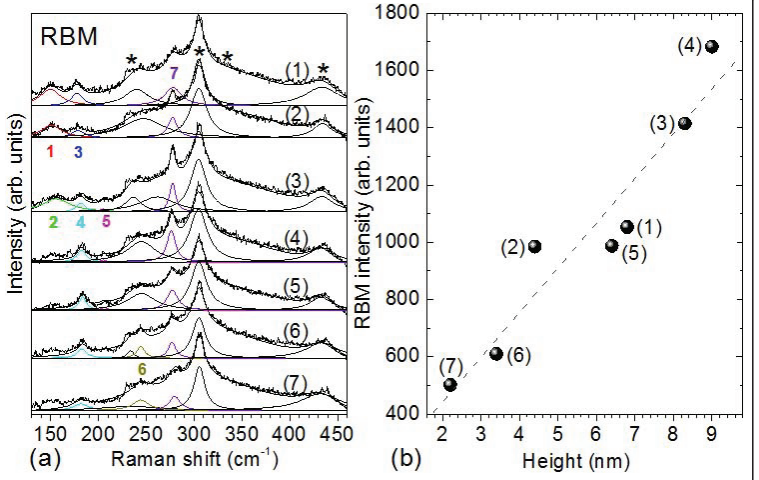
**TERS intensity as a function of the position along the SWCNT bundle and bundle height, exemplified on ***RBM_7*. **(a)** TERS spectra in the RBM region at different positions, from (1) to (7) along the SWCNT bundle. The peaks marked with stars belong to the silicon substrate. The colored numbers correspond to the RBM numbering given in Table 2. **(b) *RBM_7 ***TERS intensity as a function of the height of the bundle at the seven measurements sites.

In order to explain the appearance of different RBMs for each measured region, we will attempt to correlate each RBM with the corresponding AFM topography. In Figure 2, we see that ***RBM_7 ***is observed in the TERS spectra at each of the considered sites. This enables us to attribute its corresponding SWCNT to bundle **(*A*) **(Figure 1). Its intensity varies longwise the measured positions, becoming stronger at positions **(3) **and **(4)**, in accordance with the corresponding height profiles (Table 1) of the nanostructure **(*A*) **(Figure 1).

At positions **(1)**, **(5)**, **(2)**, and **(6) **the heights of the nanostructure are lower than at position **(4) **(Table 1, Figure 2b). Assuming a resulting increased distance between tip and nanotube, weaker intensities of ***RBM_7 ***in the TERS spectra are expected (Figure 2). The smallest intensity of ***RBM_7 ***is observed at position **(7)**, but it is still clearly visible probably because its corresponding SWCNT is oriented almost parallel to the direction of the laser polarization. Figure 2b shows the variation of the ***RBM_7 ***intensity with the nanostructure height, which can be reasonably fitted by a linear function (dashed line).

Taking into account the size of the far-field spot, we conclude that bundle **(*B*) **contributes with its far-field signal at each measured point from **(1) **to **(3)**. This nanostructure ends probably before position **(3) **or between positions **(3) **and **(4)**. The responsible RBM for this nanostructure should be ***RBM_1***, as it is present only at positions **(1) **and **(2) **(see Table 2). Furthermore we observe an RBM at 152 cm^-1 ^being very weak and broad in the far-field Raman spectra taken at positions **(1) **and **(2)**. This confirms the contribution of the confocal Raman signal to the TERS signal. The absence of ***RBM_1 ***in the TERS spectrum at position **(3) **and the appearance of ***RBM_2 ***can be explained by another, shorter tube lying on top of the tube corresponding to ***RBM_1***. This is in accordance with the AFM topography of bundle **(*B*)**. Furthermore, at positions **(1) **and **(2) **another RBM could be observed, ***RBM_3***, which is assigned to an SWCNT belonging to nanostructure **(*A*) **because it is not visible in the far-field.

Beginning with position **(3)**, a new RBM, denoted ***RBM_4***, emerges in the TERS spectra. This ***RBM_4 ***with high intensity from position **(3) **to **(6) **can be attributed to the main nanostructure ***(A) ***of the AFM topography, which has a considerable height (Figure 1, Table 2). As ***RBM_7 ***at positions **(3) **and **(4) **has considerable larger intensity than ***RBM_4***, the corresponding tube might be closer to the tip as compared to the SWCNT observed through ***RBM_4***. At positions **(5) **and **(6)**, ***RBM_4 ***has slightly larger intensity than ***RBM_7***, which indicates that the corresponding tube might be closer to the tip as compared to the SWCNT observed through ***RBM_7***. At the last position, according to the smaller height profile, the Raman intensity of ***RBM_4 ***is weak.

Moving on to ***RBM_5***, which has a weaker intensity (Figure 2, Table 2) and is observed in the TERS spectra only at positions **(3) **to **(5)**, we might attribute it to a new tube that belongs to nanostructure **(*B*) **(Figure 1) and is situated further away from the tip position. Its weak intensity cannot be associated with the nanostructure heights at the three observed positions. Moreover in the confocal Raman spectra a very weak and broad feature at ~195 cm^-1 ^was observed. Thus, ***RBM_5 ***seems to be a contribution from the far-field to the TERS spectra. At positions **(6) **and **(7)**, ***RBM_6 ***appears in the TERS measurements. The presence of this RBM can evidently be assigned to the new nanostructure **(*C*) **that emerges beginning with position **(5)**. Its higher intensity at position **(6) **is associated with its parallel orientation to the direction of the laser polarization. This SWCNT seems to be deeper situated, and by that, its intensity does not exceed the highest intensity of other observed RBMs. The weaker intensity of ***RBM_6 ***at position **(7) **appears to be due to the smallest height (Figure 1, Table 1) of the nanostructure **(*C*) **at this position.

In the confocal Raman spectra, as already mentioned, very weak and broad or no RBM at all could be observed. This might indicate that, on using the 2.33 eV excitation line, we are slightly off-resonance with the optical transitions of these carbon nanostructures.

The different RBMs observed in the TERS spectra at the seven measured positions confirm the possibility to locally characterize and to differentiate between individual SWCNTs in a bundle. Therefore, due to its lateral resolution, TERS may be successfully used in biology and medicine making possible the characterization not only at single cell level, but also at cell component level. Moreover, the enhancement through the plasmon resonances might open up new perspectives in the investigations of semiconducting materials (e.g., SiGe nanowires) and functionalized graphene.

### G-mode

**Figure 3:**
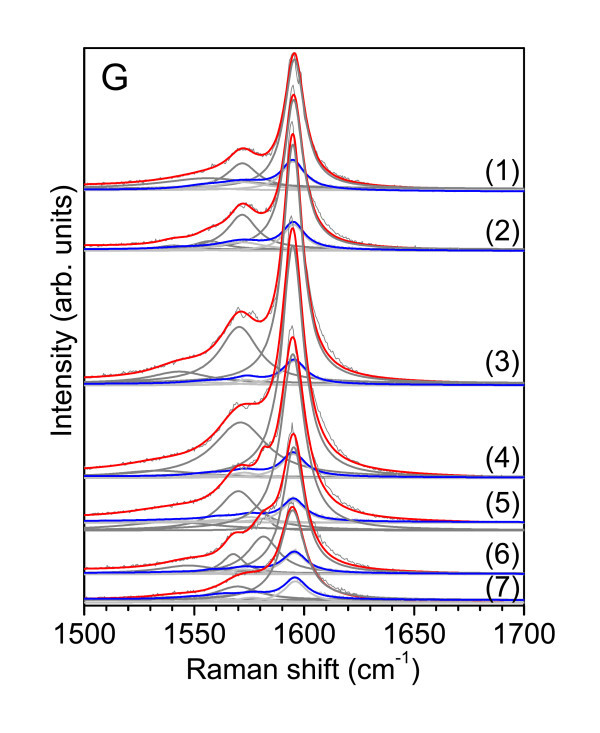
**TERS (red) and confocal Raman (blue) spectra of the G-mode at seven positions along an SWCNT bundle**. The gray and light gray peaks represent the Lorentzian fits for the TERS and confocal Raman spectra, respectively.

In Figure 3 we show the TERS and confocal Raman spectra of the G-mode at the seven chosen positions. Based on the assignment made for the RBM in the previous section, we will now discuss the dependence of the G-mode on bundle height and nanotube chiral index.

**Figure 4:**
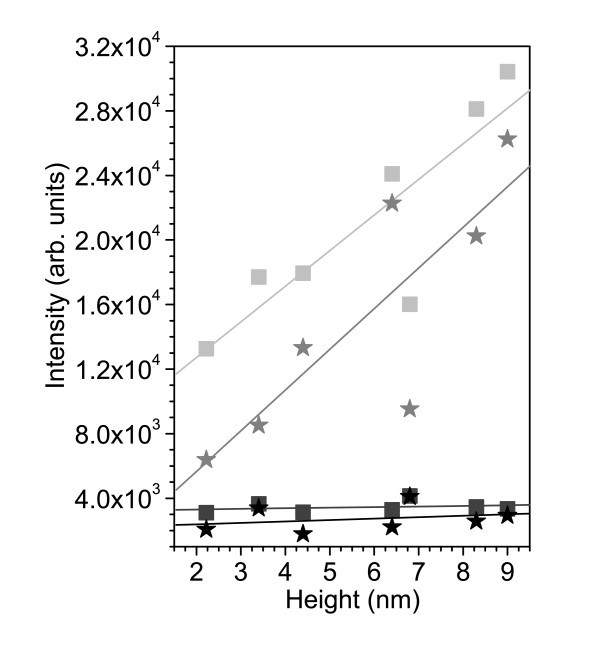
**TERS and confocal Raman intensities of the G**^**+**^**-mode (squares) and G**^-^**-mode (stars) as a function of the height of the bundle at the seven measurement sites**.

On enlarging the bundle size (i.e., higher features in the AFM topography), an increase of the G-mode intensity is to be expected. This can be partly due to the presumably smaller distance between the tip and the nanotubes, for larger bundles. Undoubtedly, however, in a larger bundle there are more carbon nanotubes contributing to the recorded Raman signal. In contrast to the RBM, the G^+ ^mode is only very slightly diameter-dependent, and the resonance window is much wider. Indeed, when plotting the G^+ ^and G^- ^intensities as a function of the bundle height, we observe an increased intensity with increasing height (see Figure 4).

Moreover, correlating the FWHM (Table 1) of the nanostructures at the measured positions, one can observe that in a larger bundle more nanotubes contribute to the TERS signal. For example, at positions **(1) **and **(5)**, where similar height profiles of bundles are observed (Figure 1, Table 1), the TERS intensities of the G^+ ^and G^- ^modes are considerably higher for the smaller bundle height [position **(5)**, Figure 4]. Owing to an FWHM of 84.82 nm at position **(5)**, in comparison to an FWHM of 61.37 nm at position **(1)**, one can argue that at position **(5) **the nanostructure is broader than at position **(1)**, and therefore, more nanotubes participate to the TERS signal. These observations emphasize the potential of the TERS technique to distinguish local vibrational properties of nanometer-sized regions.

**Figure 5:**
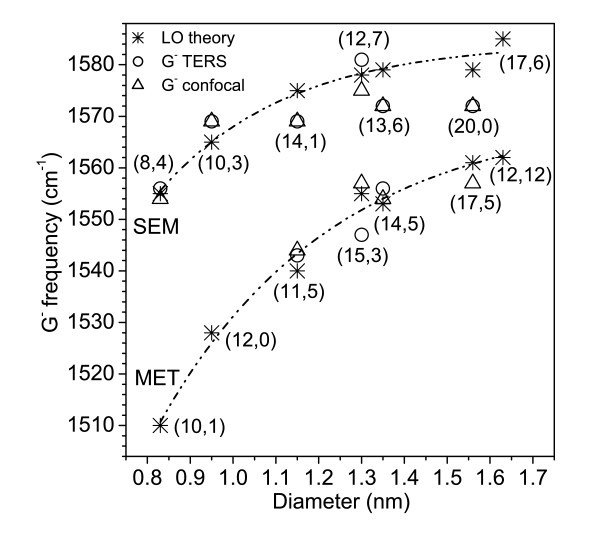
**Calculated (from ref. **[16]**) and experimental TERS and confocal Raman G^-^-mode frequencies as a function of the calculated tube diameter**. The dashed lines are drawn to guide the eye and refer to the theoretical data. SEM, semiconducting; MET, metallic.

Further on we intend to correlate the observed G^- ^peaks with the assigned RBMs. The G^+ ^and G^- ^peaks in semiconducting tubes correspond to the longitudinal (axial) and transverse (circumferential) optical vibrations, respectively, and vice versa in metallic nanotubes [14-16]. The G^- ^peak in both metallic and semiconducting nanotubes is strongly diameter-dependent. Using the assignment of RBMs to the measured positions **(1) **to **(7)**, we plot the observed G^- ^frequencies as a function of tube diameters in Figure 5.

In the TERS and confocal Raman spectra, the position of the G^+^-mode is preserved, at 1595 cm^-1^, whereas different G^-^-modes have been observed in TERS as compared to the far-field spectra. Moreover, different G^-^-modes are observed along the measured SWCNTs bundle, two for the first four positions **(1)**-**(4) **and three for positions **(5)**-**(7)**. For positions **(1) **to **(4)**, the first G^-^-peak is present at 1572 cm^-1 ^in both TERS and confocal Raman measurements, whereas the second G^-^-mode exhibits different positions (Table 3).

**Table 3:** Summary of the G- frequencies in the TERS and confocal Raman spectra along the measured bundle.

Position	Frequency (cm^-1^) in TERS			Frequency (cm^-1^) in confocal		
	**G**^-^_**1**_	**G**^-^_**2**_	**G**^-^_**3**_	**G**^-^_**1**_	**G**^-^_**2**_	**G**^-^_**3**_
**1**.	1556	1572	-	1554	1572	-
**2**.	1558	1572	-	1544	1572	-
**3**.	1543	1572	-	1557	1572	-
**4**.	1534	1572	-	1557	1572	-
**5**.	1539	1569	1581	1544	1569	1575
**6**.	1547	1569	1581	1541	1569	1575
**7**.	1547	1569	1581	1548	1569	1575

For positions **(5) **to **(7)**, a new G^- ^peak with a constant frequency of 1581 cm^-1 ^in TERS and of 1575 cm^-1 ^in confocal Raman was observed. The G^-^-mode observed previously at 1572 cm^-1 ^is now present at 1569 cm^-1^, and the third G^-^-peak has different frequencies for each position in TERS and confocal Raman, respectively (see Table 3). The appearance of various G^- ^modes at different positions along the measured bundle, and in TERS in comparison to the confocal Raman measurements, prove the effectiveness to identify the local character of this Raman feature with a spatial resolution of 100 nm by using TERS. This spatial resolution is based on the 30 nm radius of the Au-coated tip we have used and on the distances between the measured positions along an SWCNTs bundle. Thus, TERS enables the local detection and identification of optical properties of the measured SWCNTs bundle. Moreover, the detection of the different G^-^-mode in TERS can be associated with the local detection of different SWCNTs in a bundle. In this sense, based on the calculated tube diameters (Table 2) by using the observed RBM frequencies, we have correlated the observed G^-^-modes along the measured bundles with previous calculations [16]. Taking into account the calculated diameter of the tube we found good agreement with the theoretically predicted values [16] of the corresponding G^-^-modes. These were correlated with the G^-^-frequencies from our TERS and confocal Raman measurements (Figure 5).

Considering the increasing of the frequency with the increase in the diameter, one can assume that the G^-^-frequency for tube 1 (*d*_t_1 _= 1.63 nm, Table 2) is very close to the G^+^-mode frequency, and for this reason is not observed as a separate peak in the Raman spectra. However, the first G^-^-peak appears to be broader in the TERS spectra at positions **(1) **to **(3)**. Therefore, it is possible that due to this broad G^-^-mode we do not resolve the G^-^-mode corresponding to the tube 1. As for the second proposed chiral-index assignment, no experimental G^-^-frequency fits to the calculated value. As the theoretical energy *E*_ii _(Table 2) is much higher than that of the excitation energy we have used, the assignment to a metallic tube is not supported.

For tube 2 (*d*_t_2 _= 1.56 nm), taking into account the observed RBM, two different chiral indices could be considered for the assignment. However, considering the observed G^-^-frequency for both TERS and confocal Raman measurements (Figure 5) and that the observed RBM in the TERS spectra appears as a contribution from the confocal Raman signal, one can exclude the attribution to a metallic tube. Therefore the attribution to (20,0) chiral-index tube (Table 2, Figure 5) is in good agreement with both RBM and G^-^-mode frequencies assignment.

In the case of tube 3 (*d*_t_3 _= 1.35 nm), whose RBM was observed only in the TERS spectra, two chiral indices were proposed. However, one of them corresponds to a higher energy shift in comparison to the used excitation energy (Table 2). Considering the observed G^-^-frequency (Figure 5), the assignment to a semiconducting nanotube (13,6) is very likely.

The fourth observed RBM in the TERS spectra at positions **(3) **to **(7) **corresponds to tube 4 and its diameter, *d*_t_4_, varies between 1.30 and 1.32 nm. Furtheron, accounting for the observed G^-^-frequencies in the TERS and confocal Raman spectra, we can prove that two different tubes coexist in bundle **(*A*)**. Due to the excitation energy of the incoming laser at the tip apex, localized surface plasmons are excited in the apex or in the gap between the tip and the sample and therefore small shifts of the plasmon resonance can occur. These induce small shifts in the RBM frequencies and therefore tubes with slightly different diameters are observed.

For tube 5 (*d*_t_5 _= 1.15 nm), whose RBM was observed in TERS as a contribution from the far-field, also two different chiral-indices assignment were proposed. Accounting for the observed G^-^-frequencies, one can conclude that the likely assignment is to a metallic tube (11,5) (Table 2, Figure 5).

Based on the good agreement with the experimental G^-^-frequencies in the TERS and confocal Raman measurements, we have assigned tubes 6 (*d*_t_6 _= 0.95 nm) and 7 (*d*_t_7 _= 0.83 nm) to semiconducting tubes (Table 2).

Our experimental work using TERS enabled us to observe more G^-^-frequency modes than would have been possible by using conventional Raman spectroscopy. This allows us to precisely correlate the observed frequencies to the tubes' diameter, providing a more accurate assignment of the vibrational modes. This underlines once more the potential and the perspectives opened up by using the TERS technique in spectroscopically investigating nanoscaled materials.

## Conclusions

Using TERS we have probed the variation of the Raman signal of SWCNTs at seven different positions along a bundle. The TERS intensity of G-mode in carbon nanotubes is strongly dependent on the height of the bundle. Moreover, the frequency of the G^-^-mode changes from one position to the other in TERS demonstrating the strong dependence of the Raman signal on the local position along the bundle. Correlating the observed RBMs with the AFM topography we were able to identify several tubes within the observed bundles. Using TERS it is possible to differentiate between SWCNTs in bundles by using the observed RBMs. Furthermore, recording confocal Raman and TERS measurements at seven positions along a bundle we could give an accurate chiral-indices assignment, considering both, RBM and G^-^-mode frequencies.

## Abbreviations

FWHM: full widths at half maxima; HipCO: high-pressure gas-phase decomposition of CO; PL: photoluminescence; RBM: radial breathing mode; SWCNT: single-walled carbon nanotubes; TERS: tip-enhanced Raman spectroscopy.

## Competing interests

The authors declare that they have no competing interests.

## Authors' contributions

NP carried out the experiment and analyzed the data; NP and JM conceived the experiment; NP, JM, and CT discussed the results and contributed to writing the manuscript.
